# Cross sectional study of performance indicators for English Primary Care Trusts: testing construct validity and identifying explanatory variables

**DOI:** 10.1186/1472-6963-6-81

**Published:** 2006-06-28

**Authors:** Celia Brown, Richard Lilford

**Affiliations:** 1 Department of Public Health and Epidemiology, The University of Birmingham, Edgbaston, Birmingham B15 2TT

## Abstract

**Background:**

The performance of Primary Care Trusts in England is assessed and published using a number of different performance indicators. Our study has two broad purposes. Firstly, to find out whether pairs of indicators that purport to measure similar aspects of quality are correlated (as would be expected if they are both valid measures of the same construct). Secondly, we wanted to find out whether broad (global) indicators correlated with any particular features of Primary Care Trusts, such as expenditure per capita.

**Methods:**

Cross sectional quantitative analysis using data from six 2004/05 PCT performance indicators for 303 English Primary Care Trusts from four sources in the public domain: Star Rating, aggregated Quality and Outcomes Framework scores, Dr Foster mortality index, Dr Foster equity index (heart by-pass and hip replacements), NHS Litigation Authority Risk Management standards and Patient Satisfaction scores from the Star Ratings. Forward stepwise multiple regression analysis to determine the effect of Primary Care Trust characteristics on performance.

**Results:**

Star Rating and Quality and Outcomes Framework total, both summary measures of global quality, were not correlated with each other (F = 0.66, p = 0.57). There were however positive correlations between Quality and Outcomes Framework total and patient satisfaction (r = 0.61, p < 0.001) and between screening/'additional services' indicators on the Star Ratings and Quality and Outcomes Framework (F = 24, p < 0.001). There was no correlation between different measures of access to services. Likewise we found no relationship between either Star Rating or Litigation Authority Standards and hospital mortality (F = 0.61, p = 0.61; F = 0.31, p = 0.73).

**Conclusion:**

Performance assessment in healthcare remains on the Government's agenda, with new core and developmental standards set to replace the Star Ratings in 2006. Yet the results of this analysis provide little evidence that the current indicators have sufficient construct validity to measure the underlying concept of quality, except when the specific area of screening is considered.

## Background

Public services, including health, have increasingly been subjected to performance assessments, designed to fulfil the Government's "commitment to providing patients and the general public with comprehensive, easily understandable information on the performance of their local health services" [1]. Furthermore, performance assessments in health care should promote patient involvement, provide accountability and enhance patient choice [2]. However, a recent action research report has highlighted that the UK public do not like performance league tables and consider sources of information on quality as inadequate [3]. In addition, Star Ratings have induced adverse effects, such as distorted clinical priorities, bullying and reduced morale [4] in acute hospital trusts, often resulting in institutional stigma [5]. Trusts may also game with definitions of required standards, such as determining when the 8 minute ambulance call-out time actually starts [6].

Theoretically, Pringle and colleagues identify twelve methodological attributes of an ideal indicator: validity, communicable, effective, reliable, objective, available, contextual, attributable, interpretation, comparable, remediable and repeatable (see Table 1 for definitions) [7]. There is currently no Performance Indicator that fulfils all of these attributes and the existence of multiple indicators raises questions over which should be used – we return to this issue below. A further difficulty arises since some Performance Indicators are composite measures across numerous domains. While composites present a "big picture", scores are sensitive to the weighting and aggregation processes applied [8]. One essential 'acid test' considered in this paper is the construct validity of the indicator (a combination of the attributes "effective" and "comparable" used by Pringle and colleagues [7]). Construct validity implies that the indicators measure what they are intended to measure (in this case, quality). Construct validity is essential if Performance Indicators are to be used fruitfully by the public in their newly-acquired choice of providers or by regulators as a means of imposing sanctions or rewards.

This paper focuses on six Performance Indicators available in the public domain for the 303 English Primary Care Trusts (PCTs). Since no Gold Standard indicator exists, we assess the correlations between different pairs of indicators expected or hypothesised to be related. The underlying logic is that correlation is a necessary, but not sufficient, condition for construct validity. If no correlation exists, then at least one of the indicators must be an invalid measurement of a common construct. The existence of correlation is not proof of construct validity, since this requires certainty regarding causation [9]. However correlation at least suggests that whatever two correlated indicators are measuring it is the same thing: and given face validity this may be the best evidence of construct validity obtainable in circumstances where there is no Gold Standard.

Given the existence of multiple indicators, a more holistic approach to quality assessment is to consider the 'within PCT' variance across the six indicators. Differences in the relative performance of a PCT across the separate indicators may suggest that quality is not consistent across the PCT (providing that the indicators do, in fact, have construct validity). We examine 'within PCT' variances in this paper, acknowledging the reviewer who suggested this idea. Lastly, we have identified a number of features of a PCT, such as expenditure per capita, which might be correlated with the various performance measurements. We examine these in a statistical model to seek associations which might be informative.

## Methods

### Design

The analysis in this paper is a cross sectional quantitative analysis of six Performance Indicators and PCT characteristics for the 303 English PCTs.

### Data collection

The most recent data on the six Performance Indicators (Table 2) used in this analysis were downloaded from the internet during August 2005. The indicators are: Star Rating, Quality and Outcomes Framework (QOF) total, Dr. Foster Mortality index, Dr. Foster Equity index, NHS Litigation Authority (NHSLA) Risk Assessment and patient satisfaction total from the Star Ratings. The information was combined into a database using Stata v.7 (Stata Corp, Texas). Data on possible explanatory variables were then added to the database (Table 2). More detailed information on the Performance Indicators and explanatory variables can be found in Additional file 1.

### Data analysis

Relationships between Performance Indicators are assessed across two domains: pairs of indicators purporting to measure the same underlying health construct (e.g. access to services) and pairs of indicators hypothesised to be related (e.g. higher standards of care and patient satisfaction). We use both composite Performance Indicators and their components in these analyses. In identifying relationships, consideration was given to the health care setting: while a PCT may be able to foster a culture of excellence across all organisations, it may be inappropriate to expect a relationship between an indicator based solely on general practice and another based solely on hospital care. Initial assessments of relationships were undertaken using scatter diagrams if both variables were continuous with subsequent calculation of Pearson correlation coefficients if relationships appeared to be linear. For pairs including one categorical and one continuous variable, we use box and whisker diagrams and/or mean score analyses.

We apply a basic approach to assessing the 'within PCT' variance across the six Performance Indicators (based on that of Fahey and Gibberd [10]). A PCT is given one point for each indicator if the PCT's score on the indicator is better than the mean, but loses one point if the score is below the mean. No points are accrued or lost if the PCT's score is equal to the mean. For the two categorical variables, PCTs with none or one Star lose one point, those with two Stars accrue no points and those with three Stars gain one point; NHSLA Risk Assessments are scored as -1 (Level 0), 0 (level 1A) and +1 (Level 1B). We then find the total number of points for each PCT, giving a possible range of -6 (below average on all six indicators) to +6 (above average on all six indicators). An examination of the resulting score distribution provides an insight into the holistic 'quality' of the PCTs.

The effect of the five explanatory variables (Table 2) on the Performance Indicators was first explored using forward stepwise multiple regression analyses, based on ordinary least squares for continuous dependent variables and ordered logit for categorical variables. A similar method is used by Sutton and McLean in a practice-level analysis for 60 general practices in Scotland [11]. Jha and colleagues also use this approach in their analysis of US hospital performance [12]. Relationships between pairs of dependent and explanatory variables identified in the regressions were demonstrated using scatter diagrams, box and whisker diagrams, Pearson Correlation coefficients and/or mean score analyses.

## Results

### Construct validity

**Figure 1:**
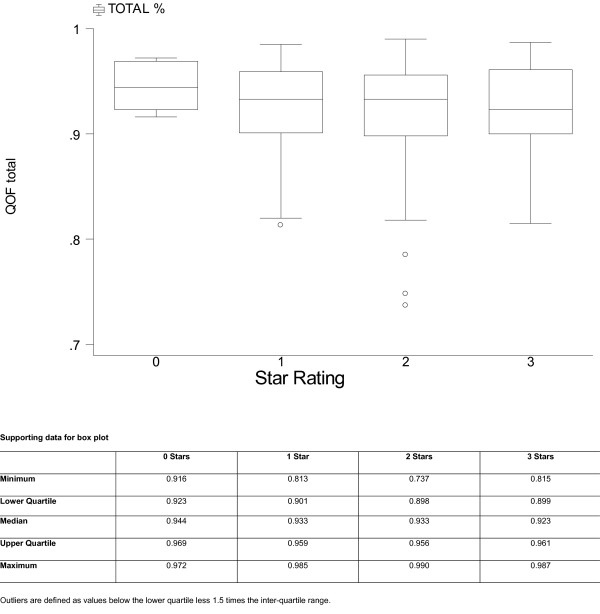
Box plot of QOF totals by Star Rating.

The first analysis investigated pairs of indicators purporting to measure the same underlying health care construct. Here, correlations would help validate the indicators, with independent measures of the same construct resulting in analogous PCT ratings. The first pair of indicators is Star Rating and QOF total, as both are composite primary care performance measures. Figure 1 shows a box and whisker diagram that analyses QOF totals for PCTs with each Star Rating. It is clear that these Performance Indicators are not related; a one-way Anova confirms no differences between QOF means across Star Ratings (F = 0.66, p = 0.57).

The Additional Services Domain on the QOF and the Improving Health category on the Star Ratings both purport to measure screening and other preventative services in general practice. Specific overlaps are cervical screening, child health surveillance and contraceptive services. Not surprisingly, there is a positive relationship between Additional Services and Improving Health assessments (Table 3).

Four indicators measure access to services: Access Bonus on the QOF, Access to Quality Services category on the Star Ratings, Equity from the Dr. Foster ratings and the Access and Waiting section on the Patient Satisfaction survey. Analysis of pairs of indicators where health care domains overlap provides insufficient evidence to suggest that these indicators are measuring the same underlying concept. The specific results of this analysis are shown in Additional file 2. It is not possible to say whether any of the access measures are 'better' than the others.

The second analysis investigated pairs of indicators measuring different health care concepts but which are hypothesised to be related. Relationships provide evidence that different indicators are valid in that they measure the general concept of 'quality' or 'performance'. First, we hypothesise that PCTs with higher Star Ratings or NHSLA Ratings would have lower hospital mortality. This is because Star Ratings provide an overall measure of PCT quality that incorporates elements of hospital care; whilst NHSLA Ratings are based on safety procedures that, if filtered down to the hospitals within a PCT's commissioning remit, should have a positive effect on the standard of care. A mean score analysis suggests that a higher Star Rating does not imply lower hospital mortality (F = 0.61, p = 0.61). PCTs with no Stars have a mean mortality ratio of 102.7, compared with 99.9 for 1 Starred PCTs, 100.2 (2 Stars) and 101.5 (3 Stars). There is a similar result for the NHSLA Rating (F = 0.31, p = 0.73): the mean mortality ratio is 99.3 for Level 0 PCTs, 100.6 for Level 1A PCTs and 100.5 for Level 1B PCTs.

**Figure 2:**
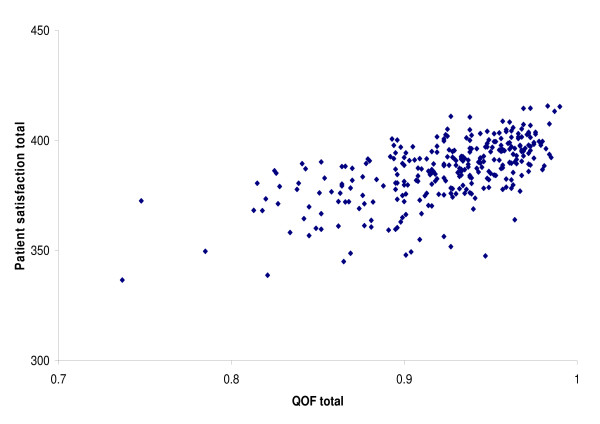
Correlation between QOF total and patient satisfaction total.

Second, we hypothesise that the better the overall standard of care in general practice (QOF total), the more satisfied are the patients. Figure 2 shows that there is a positive relationship between the quality of care and patient satisfaction (Pearson's r = 0.61, p < 0.001). The concentration of points towards the top right of the scatter plot suggests both variables are negatively skewed due to ceiling effects. One reviewer commented that the positive correlation may be driven by the outliers. Indeed, if we restrict the Pearson's r calculation to the 205 PCTs whose QOF and patient satisfaction totals both lie between the 10^th ^and 90^th ^centiles of their respective distributions, the coefficient falls to 0.38, although this is still statistically significant at p < 0.01.

#### Holistic quality assessment

**Figure 3:**
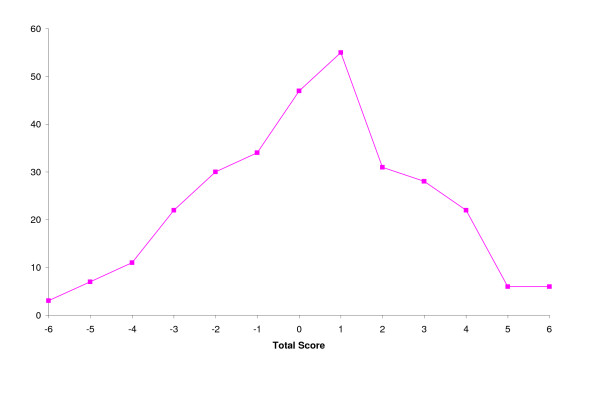
Distribution of 'holistic' quality scores across PCTs.

Our assessment of 'within PCT' variances, in which a score of -6 indicated a PCT with below average performance on all six indicators and a score of +6 a PCT with above average performance, resulted in a fairly symmetrical distribution of scores (Figure 3). 136 (45%) of PCTs had a score of -1, 0 or +1, with just three PCTs (1%) scoring -6 and six PCTs (2%) scoring +6. The distribution of the total scores in Figure 3 is consistent with the hypothesis that the individual Performance Indicators were allocated randomly and supports the finding that correlations between Performance Indicators are weak.

### Accounting for differences in PCT performance

The results for the final forward stepwise regressions for each Performance Indicator are shown in Table 4. The cumulative contribution of each explanatory variable to total R^2 ^is shown in Additional file 3.

#### Star Rating

The North East, North West and London have a greater percentage of 3 Star PCTs than the national average (50%, 40% and 35% respectively compared to 19%). PCTs in the East are the least likely to have 3 Stars, with only 5% achieving this rating. There is a weak inverse correlation between star rating and PCT expenditure per capita (F = 6.41, p < 0.001). Mean expenditure per capita for PCTs with no or one Star is €1,080 compared to €1,034 for the higher rated PCTs. There is also an inverse correlation between PCT size – the number of registered patients – and Star Rating (F = 4.51, p = 0.004). The lower rated PCTs tend to have more patients (a mean of 196,000 compared to 166,000 for PCTs with 2 or 3 Stars).

#### QOF total

**Figure 4:**
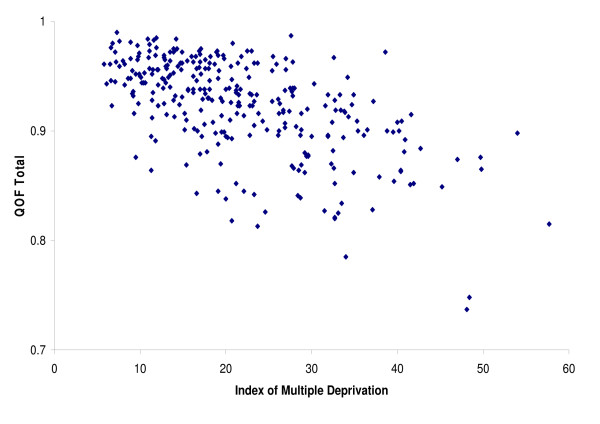
Correlation between IMD score and QOF total.

The only explanatory variable with a significant influence on a PCT's QOF total is the Index of Multiple Deprivation. There is a negative linear correlation between the Index of Multiple Deprivation and QOF, as shown in Figure 4 (Pearson's r = -0.59, p < 0.001). If the effect of outliers is removed by restricting the sample to the 201 PCTs whose Index of Multiple Deprivation and QOF scores lie between the 10^th ^and 90^th ^centiles of both distributions, the Pearson's r is reduced to -0.37 (p < 0.01). This result suggests that PCTs with the highest deprivation have the lowest QOF scores and will thus attract the least additional funding. In their analysis of practice-level data in Scotland, Sutton and McLean find that deprivation has a positive effect on scores for clinical and holistic care [11].

#### Mortality ratio

None of the explanatory variables are a good determinant of hospital mortality rates. There is evidence of small negative relationships between mortality and the number of General Practitioners per capita (Pearson's r = -0.32, p < 0.001) and PCT expenditure per capita (r = -0.28, p < 0.001).

#### Mean equity ratio

Admissions in London (mean ratio = 84.4) are less equitable than admissions in all other regions (mean ratio = 101.2; t = 5.21, p < 0.001). The equity ratio has already been adjusted for case mix and thus differences in patient demographics may not explain this result.

#### NHSLA rating

NHSLA Ratings vary by region. Compared to a national average of 29%, the percentage of PCTs with the highest rating (1B) is highest in Yorkshire and Humberside (41%) and the West Midlands (40%) and lowest in the East (15%) and North East (19%). However, the effect of region was not statistically significant in the ordered logit analysis (p > 0.05).

#### Patient satisfaction

There are two noteworthy influences on patient satisfaction. First, there is a negative relationship between Index of Multiple Deprivation and patient satisfaction (Pearson's r = -0.46, p < 0.001): i.e. poorer areas have lower satisfaction. This finding appears consistent over time, as MORI report a similar result for 2001–3 [13]. Second, patients in London report lower satisfaction than patients in other regions (with mean scores of 73% and 78% respectively; t = 9.13, p < 0.001).

## Discussion

Our analysis provided evidence of construct validity for measures of screening and preventative health care in the Star Ratings and QOF but not for different measures of access to services. At a more general level, there was no relationship between Star Rating and QOF total. Some may argue this result would be expected since it is asking a lot of PCTs to engender a level of quality across all the organisations within their commissioning remit, including both general practice and hospital care. In turn, such an argument invokes debate over the appropriateness of assessing health care performance at PCT level.

There is evidence that patients report higher satisfaction with PCTs where general practices achieve higher QOF scores. However this may be a spurious association since both QOF scores and patient satisfaction are negatively related to deprivation and hence the direction of causality is not clear. The correlations are also partly driven by the outlying PCTs in the distributions. The relationship between QOF score and deprivation contrasts with that reported by Sutton and McLean [11] which may be because our analysis is at PCT, rather than practice level, or because our analysis is based on the English, rather than the Scottish system. We also find that quality is not consistent across the six Performance Indicators in many PCTs.

Region was found to be a determinant of performance across a number of indicators. However as a further illustration of discrepancies between different Performance Indicators, patients in London receive the lowest equity in hospital admissions and report the lowest satisfaction with their care, yet have a relatively high proportion of 3 Star PCTs.

### Study limitations

This paper considered a limited number of explanatory variables: a wider range is used by Jacobs and Smith in their analysis of determinants of Star Ratings for acute hospital trusts [14]. Other possible explanatory variables could focus on organisational characteristics of PCTs and health care organisations, which is an approach taken by Sutton and McLean [11]. Alternatively, one possible variable omitted from this analysis is the age distribution of patients. Taylor et al. [13] report that the proportion of patients over 65 had a positive influence on patient satisfaction in 2002/3, and the tendency for older patients to give higher ratings of their care is noted elsewhere [15]. Consideration of ethnic differences may also explain differences in patient satisfaction, since ethnic minorities are harder to satisfy [13].

The analysis in this paper is based on a snapshot using the latest available data, requiring an assumption that measurements and explanatory variables do not change significantly over time. An alternative, longitudinal approach to measuring performance in general practice using disease-specific indicators is reported by Campbell and colleagues [16]. Based on this approach, an assessment of PCTs' ability to improve standards across different Performance Indicators may provide a different perspective to that considered here.

## Conclusion

The results in this paper cast doubts on whether any of the available Performance Indicators help the public to accurately assess the level of care received at their PCT, although Marshall and colleagues question whether patients actually value such assessments [3]. In future, it may be relevant to consider if quality can be related to health, although evidence from Canada suggests that such relationships are unlikely [9]. Performance Indicators should also provide PCTs with an incentive to improve standards of care, yet if Performance Indicators are not a valid measure of performance then scarce resources may be directed to the wrong ends.

A more detailed analysis of multi-faceted indicators, to identify outliers on particular components of the indicators, may help PCTs prioritise areas for improvement. This analysis could be based on the methods of Gibberd and colleagues [17] and we are currently planning a study of this nature based on the 2006 Health Check data, once these data become available.

Given that a PCT's responsibilities are so multi-faceted, health care performance may not be best measured at PCT level. QOF totals, mortality and mean equity have been aggregated to PCT level from either general practice or hospital level and the aggregation process is likely to mask individual differences in performance across the PCT. A possible solution is for PCT level indicators to be focused only on aspects of care within the direct control of the PCT, rather than indirect aspects that can only be assessed in the care organisations commissioned by the PCT.

The Star Rating scheme is currently being revised to include monitoring of both core standards and progress towards developmental targets, which will introduce an improvement element to the existing purposes of quality assurance and accountability [18,19]. We await an evaluation of this Annual Health Check in due course, including an assessment of the validity of the indicators.

## List of abbreviations

NHSLA: National Health Service Litigation Authority

PCT: Primary Care Trust

QOF: Quality and Outcomes Framework

## Authors' contributions

CB and RL developed the study. CB undertook the data gathering and analysis. CB and RL drafted the paper.

## Competing interests declaration

RL and CB have been invited to/have attended Safety Expert Reference Group meetings at the Healthcare Commission regarding the Safety developmental standards within the Annual Health Check.

## Pre-publication history

The pre-publication history for this paper can be accessed here:



## Supplementary Material

Additional data file 1 Supplementary information on performance indicators. Provides more information on each performance indicator used in the analysis, with its source. Click here for file

Additional data file 2 Comparisons of different measures of access to services. Provides 2-way comparisons of different combinations of measures of access to services across PCTs. Click here for file

Additional data file 3 Forward step-wise regressions accounting for differences in PCT performance. Provides detail of the order and R^2^/puesdo R^2 ^for each step in the regression analysis. Click here for file

## Figures and Tables

**Table 1:** Descriptions of attributes of ideal quality indicators

**Attribute**	**Description**
Validity	Meeting the standard seen as better quality
Communicable	Relevance of measure can be explained
Effective	Indicator measures what it purports to measure
Reliable	Data are complete, accurate, consistent and reproducible
Objective	Data are independent of subjective judgement
Available	Data are available quickly/routinely with minimal cost or effort
Contextual	Indicator is context free
Attributable	Performance on an indicator can be attributed to the relevant individual or team
Interpretation	Indicator should reflect health needs, capacity, structures or performance
Comparable	Indicator should be comparable to a gold standard
Remediable	Poor performance on an indicator can be remedied
Repeatable	Indicator should be sensitive to improvement

Attributes and descriptions taken from Pringle et al. [7].

**Table 2:** PCT performance indicators and explanatory variables

**Performance Indicators**				**Explanatory Variables**			
Performance Indicator	Year of data	Mean	SD	Explanatory variable	Year of data	Mean	SD

Star Rating	2005	0 stars – 7		FTE GPs per 100,000 population	2005	58.1	7.6
		1 star – 81					
		2 stars – 157					
		3 stars – 58					
Quality and Outcomes Framework (QOF) (%)	2005	92.3	4.3	Expenditure per capita (€)	2003–4	1,047	86.7
Dr Foster – Mortality (Index)	2002–4	100	8.6	Number of patients	2005	174,000	68,000
Dr Foster – Equity (Index)	2001–4	100	17.7	Index of Multiple Deprivation (%)	2004	21.5	10.1
NHSLA	2005	Level 0 – 34		Region	N/A	N/A	N/A
		Level 1A – 181					
		Level 1B – 88					
Patient Satisfaction (%)	2005	77.3	2.8				

Notes:

QOF data based on 302 PCTs

Dr Foster Equity data based on 299 PCTs: mean of equity indices for heart by-pass and hip replacement procedures

NHSLA: NHS Litigation Authority

NPCRDC: National Primary Care Research and Development Centre

Patient Satisfaction data taken from the Star Ratings

**Table 3:** Relationship between Improving Health rating (Star) and Additional Services score (QOF)

**Improving Health Rating**	**Number of PCTs**	**Additional Services Mean**	**Standard Deviation**	**% of PCTs scoring 100% on Additional Services**
Low	50	92.8%	7.3	42%
Medium	80	96.4%	5.3	66%
High	172	98.3%	3.8	83%

One-way ANOVA between Additional Services means; F = 24.0, p < 0.001.

**Table 4:** Final forward stepwise regression results

**Star Rating (ordered logit)**		
Pseudo R^2 ^= 0.1154		
**Explanatory Variable**	**Coefficient**	**Z-value**

GPs per 100,000 population	-0.021	-1.28
Expenditure per capita	-0.001	-0.69
Number of patients	0.000	-1.90
Index of Multiple Deprivation	0.020	1.50
*Region (control = London):*		
North West	2.626	5.08
North East	3.021	4.62
Yorkshire and Humberside	0.932	1.72
West Midlands	1.684	3.02
East Midlands	1.488	2.63
East	0.571	1.08
South East	0.694	1.39
South West	1.531	2.72

**QOF Total**		
R^2 ^= 0.4527		

**Explanatory Variable**	**Coefficient**	**t-value**

GPs per 100,000 population	0.001	2.40
Expenditure per capita	0.000	-0.23
Number of patients	0.000	-0.56
Index of Multiple Deprivation	-0.003	-10.96
*Region (control = London):*		
North West	0.029	3.11
North East	0.462	3.86
Yorkshire and Humberside	0.210	2.11
West Midlands	0.008	0.81
East Midlands	0.021	1.94
East	0.008	0.83
South East	0.239	2.57
South West	0.342	3.34
Constant	0.928	26.04

**Mortality Ratio**		
R^2 ^= 0.3242		

**Explanatory Variable**	**Coefficient**	**t-value**

GPs per 100,000 population	-0.232	-3.82
Expenditure per capita	-0.024	-4.41
Number of patients	0.000	0.55
Index of Multiple Deprivation	-0.125	-2.54
*Region (control = London):*		
North West	3.884	2.05
North East	-0.227	-0.09
Yorkshire and Humberside	-1.715	-0.86
West Midlands	9.932	4.79
East Midlands	4.290	2.02
East	6.001	2.99
South East	4.316	2.32
South West	3.416	1.67
Constant	138.0	19.36

**Mean Equity Ratio**		
R^2 ^= 0.1448		

**Explanatory Variable**	**Coefficient**	**t-value**

GPs per 100,000 population	-0.370	-2.62
Expenditure per capita	0.009	0.70
Number of patients	0.000	-0.17
Index of Multiple Deprivation	-0.025	-0.22
*Region (control = London):*		
North West	22.21	5.09
North East	18.89	3.42
Yorkshire and Humberside	16.70	3.63
West Midlands	15.03	3.14
East Midlands	11.61	2.37
East	17.02	3.65
South East	10.55	2.44
South West	22.47	4.75
Constant	97.71	5.93

**NHSLA Rating (ordered logit)**		
Puesdo R^2 ^= 0.0432		

**Explanatory Variable**	**Coefficient**	**Z-value**

GPs per 100,000 population	0.001	0.06
Expenditure per capita	-0.001	-0.64
Number of patients	0.000	1.68
Index of Multiple Deprivation	-0.026	-1.91
*Region (control = London):*		
North West	0.892	1.69
North East	-0.416	-0.60
Yorkshire and Humberside	0.926	1.66
West Midlands	0.862	1.50
East Midlands	-0.346	-0.58
East	-0.467	-0.82
South East	0.046	0.09
South West	0.401	0.70

**Patient Satisfaction Total**		
R^2 ^= 0.5155		

**Explanatory Variable**	**Coefficient**	**t-value**

GPs per 100,000 population	0.327	3.93
Expenditure per capita	0.018	2.37
Number of patients	0.000	-1.90
Index of Multiple Deprivation	-0.649	-9.68
*Region (control = London):*		
North West	22.56	8.72
North East	24.51	7.49
Yorkshire and Humberside	19.92	7.31
West Midlands	15.53	5.48
East Midlands	21.94	7.57
East	12.07	4.39
South East	14.19	5.57
South West	21.29	7.59
Constant	349.48	35.86
