# Supplementary material for: Cross sectional study of performance indicators for English Primary Care Trusts: testing construct validity and identifying explanatory variables
Source: BMC Health Serv Res. 2006 Jun 28;6:81. doi: 10.1186/1472-6963-6-81 (PMC1526428; doi:10.1186/1472-6963-6-81)
Supplement: Additional data file 1 — Supplementary information on performance indicators. Provides more information on each performance indicator used in the analysis, with its source. [file 1472-6963-6-81-S1.doc]

**Additional Data 1**

*Supplementary information on performance indicators*
