# Supplementary material for: Cross sectional study of performance indicators for English Primary Care Trusts: testing construct validity and identifying explanatory variables
Source: BMC Health Serv Res. 2006 Jun 28;6:81. doi: 10.1186/1472-6963-6-81 (PMC1526428; doi:10.1186/1472-6963-6-81)
Supplement: Additional data file 2 — Comparisons of different measures of access to services. Provides 2-way comparisons of different combinations of measures of access to services across PCTs. [file 1472-6963-6-81-S2.doc]

**Additional Data 2**

*Comparisons of different measures of access to services*

1. Access Bonus (QOF) and Access to Quality Services (Star)

| AQS Rating | Number of PCTs | AB Mean | AB Standard Deviation | % of PCTs scoring 100% on AB |
| --- | --- | --- | --- | --- |
| Low | 50 | 96.4% | 5.9 | 52% |
| Medium | 95 | 97.4% | 4.0 | 59% |
| High | 157 | 97.8% | 3.2 | 61% |

One-way ANOVA between AB means; F=2.20, p=0.1124.

1. Equity (Dr. Foster) and Access to Quality Services (Star)

| AQS Rating | Number of PCTs | Equity Mean | Equity Standard Deviation | Equity Minimum | Equity Maximum |
| --- | --- | --- | --- | --- | --- |
| Low | 50 | 94.0 | 17.0 | 62.8 | 168.5 |
| Medium | 94 | 100.4 | 17.4 | 58.7 | 171.5 |
| High | 155 | 100.7 | 17.9 | 42.5 | 172.4 |

One-way ANOVA between Equity means; F=2.88, p=0.058.

1. Access Bonus (QOF) and Access and Waiting (Patient Satisfaction)

Due to the ceiling effect on the Access Bonus variable, comparing these indicators is problematic. 59% of PCTs score the maximum 100% for Access Bonus, with the lowest score being 75%. A Scatter diagram of these two indicators simply reveals the significance of the ceiling effect, with no evidence of a positive linear relationship, hence making a correlation coefficient inappropriate.

1. Access Bonus (QOF) and Equity (Dr. Foster)

This combination is not considered as the first indicator is based in general practice and the second in hospital.

1. Access and Waiting (Patient Satisfaction) and Equity (Dr. Foster)

Not considered as in the first indicator is based in general practice and the second in hospital.

1. Access to Quality Services (Star) and Access and Waiting (Patient

Satisfaction).

Not considered as the patient satisfaction survey forms part of the Access to Quality Services category on the Star Ratings.
