# Supplementary material for: Cross sectional study of performance indicators for English Primary Care Trusts: testing construct validity and identifying explanatory variables
Source: BMC Health Serv Res. 2006 Jun 28;6:81. doi: 10.1186/1472-6963-6-81 (PMC1526428; doi:10.1186/1472-6963-6-81)
Supplement: Additional data file 3 — Forward step-wise regressions accounting for differences in PCT performance. Provides detail of the order and R2/puesdo R2 for each step in the regression analysis. [file 1472-6963-6-81-S3.doc]

**Additional Data 3**

*Forward step-wise regressions accounting for differences in PCT performance*

| **Independent Variable** | **Dependent Variable** | **Regression Type** | **R^2^/Puesdo R^2^** |
| --- | --- | --- | --- |
| Star Rating | FTE GPs per 100,000 population | Ordered Logit | 0.0032 |
|  | Expenditure per capita |  | 0.0192 |
|  | Number of patients |  | 0.0184 |
|  | Index of Multiple Deprivation |  | 0.0370 |
|  | Region |  | 0.1154 |
| QOF Total | FTE GPs per 100,000 population | OLS | 0.0111 |
|  | Expenditure per capita |  | 0.0476 |
|  | Number of patients |  | 0.0859 |
|  | Index of Multiple Deprivation |  | 0.3892 |
|  | Region |  | 0.4527 |
| Mortality Ratio | FTE GPs per 100,000 population | OLS | 0.1050 |
|  | Expenditure per capita |  | 0.1566 |
|  | Number of patients |  | 0.1592 |
|  | Index of Multiple Deprivation |  | 0.2034 |
|  | Region |  | 0.3242 |
| Mean Equity Ratio | FTE GPs per 100,000 population | OLS | 0.0124 |
|  | Expenditure per capita |  | 0.0132 |
|  | Number of patients |  | 0.0345 |
|  | Index of Multiple Deprivation |  | 0.0346 |
|  | Region |  | 0.1448 |
| NHLSA Rating | FTE GPs per 100,000 population | Ordered Logit | 0.0000 |
|  | Expenditure per capita |  | 0.0001 |
|  | Number of patients |  | 0.0052 |
|  | Index of Multiple Deprivation |  | 0.0077 |
|  | Region |  | 0.0432 |
| Patient Satisfaction | FTE GPs per 100,000 population | OLS | 0.0308 |
|  | Expenditure per capita |  | 0.0523 |
|  | Number of patients |  | 0.1526 |
|  | Index of Multiple Deprivation |  | 0.3201 |
|  | Region |  | 0.5155 |
